# FedDNA: Federated learning using dynamic node alignment

**DOI:** 10.1371/journal.pone.0288157

**Published:** 2023-07-03

**Authors:** Shuwen Wang, Xingquan Zhu

**Affiliations:** Department of Electrical Engineering and Computer Science, Florida Atlantic University, Boca Raton, Florida, United States of America; TU Wien: Technische Universitat Wien, AUSTRIA

## Abstract

Federated Learning (FL), as a new computing framework, has received significant attentions recently due to its advantageous in preserving data privacy in training models with superb performance. During FL learning, distributed sites first learn respective parameters. A central site will consolidate learned parameters, using average or other approaches, and disseminate new weights across all sites to carryout next round of learning. The distributed parameter learning and consolidation repeat in an iterative fashion until the algorithm converges or terminates. Many FL methods exist to aggregate weights from distributed sites, but most approaches use a *static node alignment* approach, where nodes of distributed networks are statically assigned, in advance, to match nodes and aggregate their weights. In reality, neural networks, especially dense networks, have nontransparent roles with respect to individual nodes. Combined with random nature of the networks, static node matching often does not result in best matching between nodes across sites. In this paper, we propose, FedDNA, a *dynamic node alignment* federated learning algorithm. Our theme is to find best matching nodes between different sites, and then aggregate weights of matching nodes for federated learning. For each node in a neural network, we represent its weight values as a vector, and use a distance function to find most similar nodes, *i.e.*, nodes with the smallest distance from other sides. Because finding best matching across all sites are computationally expensive, we further design a minimum spanning tree based approach to ensure that a node from each site will have matched peers from other sites, such that the total pairwise distances across all sites are minimized. Experiments and comparisons demonstrate that FedDNA outperforms commonly used baseline, such as FedAvg, for federated learning.

## Introduction

Federated Learning (FL), originally proposed in 2016 [1], is a learning paradigm which builds machine learning models based on datasets distributed across multiple sites/devices in order to protect privacy and prevent data leakage. While traditional machine learning methods are typically trained based on centralized data, using FL provides a feasible way to develop models that can keep all the training data on distributed devices and update model parameters using immediate aggregation.

As data collection and analytics are becoming increasingly popular, protecting data privacy and safety is becoming a major concern for business, government, and nearly all sections of human society. By deploying FL, each participant in the model training process can build one model together without sharing data, naturally results in data privacy protection. Traditional machine learning methods need to concentrate training data in a certain machine or a single data center, which means in order to meet the gradually increasing data level, it is necessary to continuously add machines and build infrastructure. Such method not only greatly increases the cost but also hinders the efficiency building models. In contrast, FL allows all the needed data stay in their local places without the need to build specific data center to aggregate them, at the same time, each part of the data will be used to develop the model. Such efficient characteristic enables Federated Learning to be widely used in multiple areas especially in the healthcare domain.

### Federated learning in healthcare

The shift from written health records to electronic health records has been instrumental in driving the use of patient data to improve the healthcare industry. The adoption of electronic health records enables health care professionals to disseminate knowledge across all sectors of health care, which in turn helps to reduce medical errors and improve patient care and satisfaction. However, as mentioned previously, adequate medical data sets are difficult to obtain. However, in order to capture the subtle relationships between disease patterns, socioeconomic and genetic factors, and complex and rare cases, exposing the model to different cases is critical. FL is able to address this issue by enabling the distributed training of machine learning models using remotely hosted datasets without the need to accumulate data and therefore compromise the data privacy [2–7].

While FL is capable of making use of data across different sites/institutions, there are still several data acquisition issues which can cause bias during model develop process. First of all, due to data privacy limitation, the Health Insurance Portability and Accountability Act (HIPAA) has set up regulations for healthcare organizations to manage and safeguard personal information and address their risks and legal responsibilities in relation to processing personal patients data [8]. This leads to strict data share policies of each healthcare provider, which, limits the amount of available data source. Another issue is that there exist hospital speciality gaps between different hospitals, in other words, healthcare providers might focus on several particular diseases treatment instead of performing general hospitalization. In this case, there are big chances where FL models trained across all different disease focus datasets will perform predictions with certain disease-specific bias. In addition, biases also exist when patients demographic characteristics differ. Different income groups, age groups, genders, and geographical locations and living environments will all affect the overall patient characteristics that admitted to different regional hospitals, thus, data bias can also be observed in such kind of dissimilarity. Therefore, it is essential to reduce all the above biases when we try to develop a federated learning model to make crucial medical predictions. We aim to design a novel federated learning model that can take this kind of bias into consideration at the first step where node weight aggregation takes place.

### Federated learning uniqueness and limitation

Despite the fact that use of traditional machine learning techniques (TML) in combination with electronic health records (EHR) is gaining popularity as a means to extract knowledge that can improve decision-making processes in healthcare, they usually require the training of high-quality learning models based on diverse and comprehensive datasets that are difficult to obtain due to the sensitivity of medical data from patients. Meanwhile, although distributed machine learning [9] has addressed parallel computing in handling large scale data, these methods are often designed to tackle the data volumes using frequent data exchange. In addition, switch learning models are often prohibitively expensive/inconvenient, making it difficult for end users to try/implement different learning algorithms. On the contrast, FL enables devices to collaboratively learn shared predictive models while keeping all training data on-device, decoupling the power of machine learning from the need to store data in the cloud. This goes beyond using native models to make predictions on mobile devices and also brings model training to the device.

**Fig 1:**
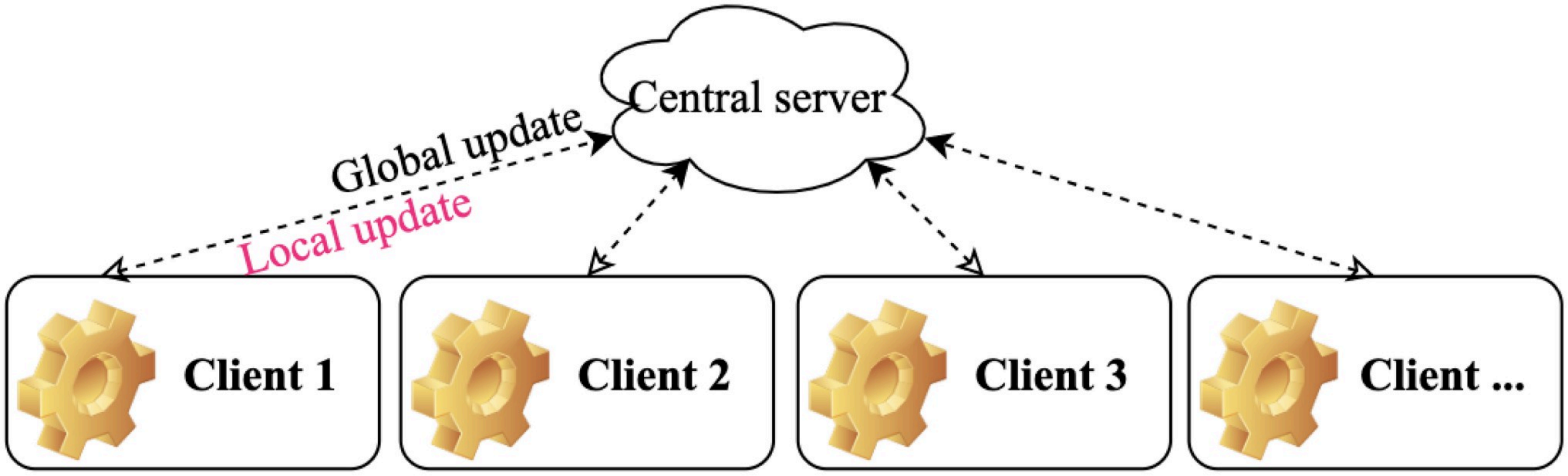
A conceptual view of the FL framework. The local update (downstream) and global update (upstream) are carried out iteratively to ensure models trained using local data are aggregated at central server, and then dispatched to distributed sites.

Table 1 summarizes the main difference between federated learning, traditional machine learning methods, and distributed machine learning methods. In summary, the inherent advantage of federated learning is that is allows flexible modeling training and continuous learning on end-user devices while ensuring no end-user data leaves the device. Fig 1 shows how FL works. Global model *M* is downloaded from the central server to each client when it comes to training the model, after which the downloaded model is trained by each client using their own dataset. Once the training process is completed, each client needs to update their updated training parameters to the central server and the central server would aggregate the learnt parameters (parameter aggregation) and pass the aggregation results to the global model, therefore, one update for the global model is accomplished and this process is called Global update. Once global update is finished, model parameters will be passed from the global model to each local model for Local update, where clients’ model parameter will be updated with the new aggregated model weights to start a new round training. [4].

**Table 1:** Comparison between Federated Learning (FL), traditional machine learning (TML), and distributed machine learning (DML) algorithms. DML methods are commonly data driven (DML_*d*_) or computing driven (DML_*c*_). Data driven methods (DML_*d*_) mainly try to learn from large volume distributed data, whereas computing driven methods (DML_*c*_) aim to parallelize computing in learning from centralized data. *Computing framework\* refers to the whole eco-system for learning, and *model switch* refers to easiness of switching a new learning model.

Method	Data location	Main computing	Computing framework	Data exchange	Main Challenge	Privacy protection	Model switch
TML	Centralized	Data center	Very restrictive	Yes	Model performance	Low	Very restrictive
DML_*c*_	Centralized	Data center	Restrictive	Yes	Data volume	Low	Very restrictive
DML_*d*_	Distributed	Local	Flexible	Yes	Data volume	Medium	Restrictive
FL	Distributed	Local	Very flexible	Prohibited	Data protection	High	Flexible

Parameter aggregation is one of the most important steps of the federated learning. Among all existing methods, Federated Averaging (FedAvg) is the most commonly used method. Eq 1 summarizes the global weight values **w** updating of FedAvg in each training round *t*, in which *k* is the client index, *K* means the total number of clients, *n* is the total number of instances and *n*_*k*_ is the local data examples for each client [1]. Overall, Eq 1 indicates that the global weight vector **w** is the weighted average of weight values obtained from local clients. In this paper, a bold-faced symbol denotes a vector or a high dimensional vectors (*e.g.* a matrix).

$$\begin{array}{c} w_{t+1}=\sum_{k=1}^{K}\frac{n_{k}}{n}w_{t}^{k} \end{array}$$ (1)

Recently, other weight aggregation methods have also been proposed in FL. For example, anomaly score of each client is taken into consideration to detect abnormal client behavior, thus, clients will not contribute equally when global model updates the weight values, the majority of those novel methods are still based on FedAvg [10, 11]. Even though this method is widely used and has been proved with good prediction performance [12, 13], due to the nature of hidden layers in deep learning neural networks, we can clearly observe that this method manually forces weight aggregations between neurons located at the exact same location (*i.e.*, same layer and same node index) of two networks. However, when training two same-structured deep learning networks *N*_*A*_ and *N*_*B*_, even they are given the same input, neurons at the same location of the two networks do not always give the same update. In other words, certain property of the input (or the same instance) may trigger the most significant activation to the *i*-th node of *N*_*A*_, but same instance may triger the most significant activation to the *j*-th node of *N*_*B*_. Meaning that same instance responds differently for the same lactation nodes between two networks.

**Fig 2:**
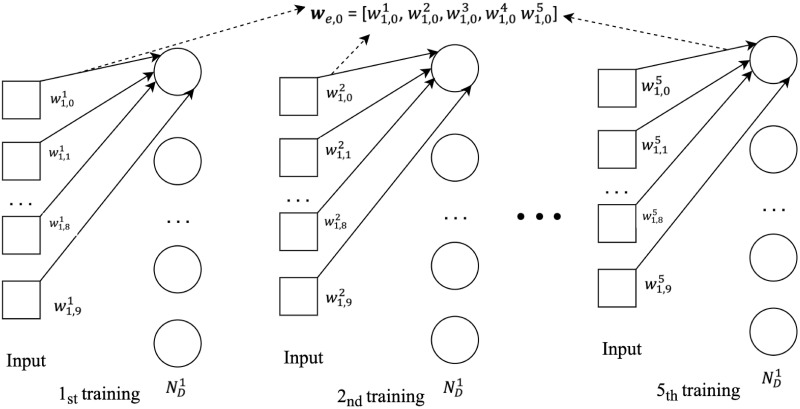
A conceptual view of node weight variance calculation. Five neural networks with the same architecture are trained using same training sample. The first hidden layer nodes are trained with the same input features and the first node is chosen to calculate the node variance.

**Fig 3:**
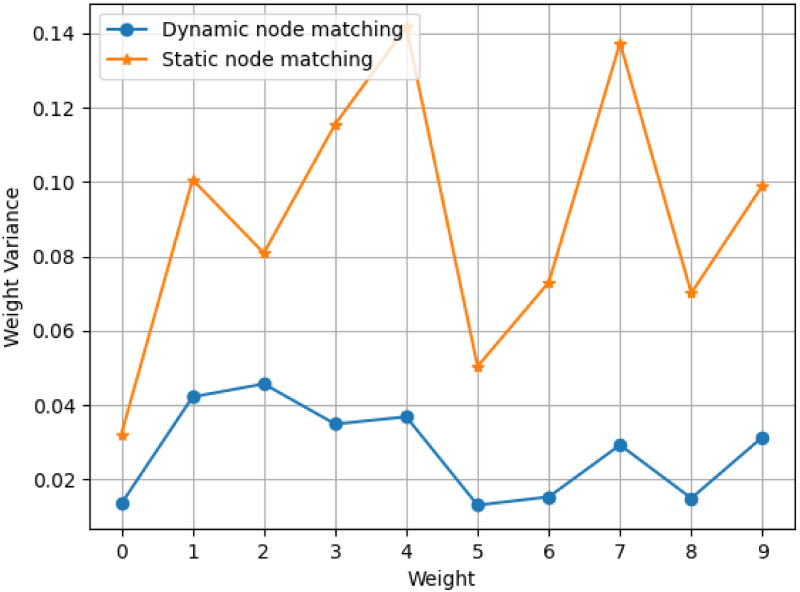
Comparisons of weight variance between two weight matching methods, *Static node matching* vs. *Dynamic node matching* (proposed). The *x*-axis denotes the neuron node ID of the first hidden layer, consisting of 10 neurons, of a neural network. The network was trained five times till convergence, using same training data. The *y*-axis denotes the variance of the weight values of each of the hidden nodes (Larger variance mean the neuron weights are more unstable across different training times, even for the same feature dimension of the same neuron).

In order to demonstrate the above hypothesis, we create a simple dense neural network *N*_*D*_ with one input layer, two hidden layers and one output layer. One dataset with 10 features is fed into *N*_*D*_. For the *i*^*th*^ node in the first hidden layer $N_{D}^{1}$, there will be 10 weight values $\left\{w_{i,0}^{1},w_{i,1}^{1},\ldots ,w_{i,9}^{1}\right\}$ corresponding to the 10 input features (the superscript denotes the first trained network). After we train *N*_*D*_ from scratch for five times with the exactly same dataset, a node *e* is randomly chosen from all five networks (with the same node index), from which we will get 10 weight vectors of {**w**_*e*,0_, **w**_*e*,1_, …, **w**_*e*,9_} in which $w_{e,0}=\left[w_{i,0}^{1},w_{i,0}^{2},w_{i,0}^{3},w_{i,0}^{4},w_{i,0}^{5}\right]$ represents all five trained weight values corresponding all five networks’ first indexed node and first feature dimension as shown in Fig 2. After that, we calculate the variance of **w**_*e*,0_, and repeat the same for all 10 nodes. Fig 3 reports the variance of the weight values across all five trained network. The high weight variance in Fig 3 concludes that weight aggregation by static node matching will not only add uncertainty to model performance, but also will hinder the practical application of Federated Learning in industry.

In this paper, we aim to design a dynamic node matching method, FedDNA, to aggregate weight values in each round based on a neuron-distance method, in which neuron distances across all the clients are calculated after each client completes training the model parameters with their own data. After that, the closest neurons are matched to calculate their average weight values as new parameter for the global model. Fig 3 reports weight variance of the matched nodes trained using same setting as the static node matching. The results show that dynamic node matching provides much smaller weight variance across all nodes of different networks. The advantage of reducing variance is that it allows nodes with similar behaviors to be aggregated for weight averaging. This potentially results in stable and improved federated learning performance.

In summary, the main contribution of the proposed research is summarized as follows:

- **Dynamic node alignment:** We propose a new dynamic node alignment framework, FedDNA, for weight aggregation in federated learning. Instead of using fixed node index to match nodes across different sites, FedDNA finds the best matching nodes based on node weight values, such that nodes, of the same layer, with the most similar response to the input are considered as one new node for next round training.
- **Fast node alignment:** To increase node aliment speed, we propose a Minimum Spanning Tree (MST) based method to find global optimal matching nodes across all sites.
- **Alignment and frozen:** In each training process, after finding the matching nodes at the very beginning, node matching will be frozen and federated average will be used for the rest of training rounds. By doing this, we can ensure the matching nodes orders which will not be disturbed by subsequent training.

## Related work

### Dynamic node alignment

Conventional FedAvg is proposed to strictly average local node weights in order to update the global model parameters, which has inspired researchers to try to come up with more flexible and reasonable node alignment ideas. A new federated learning algorithm called Fed2 incorporating a feature alignment strategy in order to enable local models to align feature representations with a global model by introducing a feature projection layer is proposed and validated [14]. In this method, the authors introduce a feature projection layer, which is able to map the features extracted by the local models to a common feature space that is aligned with the global model. During training, the local models use this layer to project their features into the common feature space, where the distances between the projected features of both the local models and global models can be minimized. A method for aligning two models in federated learning by matching neurons that perform similar functions is proposed, in which the matching is done by projecting the local update onto the tangent space of the reference model, which ensures that the update is aligned with the reference model’s geometry. The weights used in the averaging are proportional to the cosine similarity between each local update and the reference model. This weighting scheme gives more weight to updates that are more aligned with the reference model and less weight to updates that are less aligned, which helps to improve the overall convergence speed and accuracy of the algorithm [15]. Based on the previous approach, a new approach Multi-Center Federated Learning that aims to improve personalization by clustering clients based on their data distribution is proposed. The multi-center aggregation mechanism this approach involves aggregating the local models from multiple centers to obtain a global model. Each center trains its own local model based on the data of the clients in its cluster. The local models are then aggregated by exchanging information between the centers to improve the global model [16].

### Federated learning node aggregation

As more attention to data privacy protection are being payed, Federated Learning has become one of the most popular areas. Certain number of studies have also proposed creative approaches related to weight update in Federate Learning. LoAdaBoost FedAvg is proposed to achieve higher model prediction accuracy on distributed intensive care data, in which local models with a high cross-entropy loss were further optimized before model averaging on the server [17]. Federated-Autonomous Deep Learning (FADL) is designed to update global model by training part of the model using all data sources in a distributed manner while the rest of the model is trained with data from specific data sources [18]. When it comes to IID data, Haddadpour and his colleagues introduce a framework called federated averaging with compression (FedCOM), which the global model is decided not only by the update by the average of all clients’ training results, but also determined by the previous global model [19]. A model poisoning attack is proposed to perform adversary controlling of a small number of malicious clients in order to ensure weight updates are not being poisoned by those clients [20]. Similarly, abnormal clients are observed at the server side in the proposed detection-based method to timely detect unusual clients behaviors to prevent abnormal server model updates, in which a low dimension substitute of the weight vectors is created for anomaly detection [10]. A novel FL approach using mutual information (MI) which weight updates are reformulated by minimizing the MI between the local model and the aggregate model and adopting a negative correlation learning (NCL) strategy on the client side. The convergence of this algorithm theoretically is proved by experiments on MNIST, CIFAR-10, ImageNet and clinical MimIC-III datasets [21]. A hierarchical federated edge learning framework is proposed in order to solve the optimization issue of device scheduling and resource, in which immediate hospitals are in charge of part of model aggregation work [22]. To prevent unreliable updates from untrusted devices, a new concept called reputation has been introduced in the context of FL-enabled healthcare systems. This reliable device choice plays an important role in mitigating multiple security attacks [23]. A new optimization algorithm for federated learning that leverages over-the-air computation is proposed, in which the authors aim to improve the convergence rate and accuracy of federated learning by introducing a new learning rate optimization algorithm that takes into account the channel conditions and interference between the devices in a federated learning network. This new algorithm uses a feedback mechanism to adjust the learning rate dynamically, which improves the convergence rate and accuracy of the federated learning model [24]. Federated Loss-Weighted Averaging (FedLWA) scheme is a key component in an unsupervised recurrent federated learning (URFL) algorithm to enhance the parameter aggregation, in which each edge device calculates a weight based on the loss function of its local model during training [25].

Local-update SGD method which is a technique running stochastic gradient descent (SGD) parallel on various workers and the sequences will only be updated once in a while has been proved with faster convergence and less communication cost for Federated Learning [26]. Two ideas based on SGD are tested and proved convergence. The first strategy is *local steps* and the second idea aims to share certain information and do local computations. The results show higher convergence speed and more efficient communication on Federated Learning settings using these two SGD based methods [27]. Several gap settings are emphasized between the upper and lower limits of optimization associated with federated learning, particularly for “intermittent communication graphs” that capture local SGD methods but whose convergence rates are not known to match the corresponding lower limits [28]. Computational heterogeneity generated by Local-update SGD in Federated Learning is analyzed and one solution to the client fragmentation problem is to fix the number of local updates at a particular point, but allow clients to update the global model fashion asynchronously or without locking [29]. Researchers assume that all clients are involved, at the same time using batch gradient descent on all clients may be better than using stochastic gradients [30]. Li et al. [31] studied SGD convergence in a more realistic environment for joint averaging, involving only a subset of customers per round. To ensure convergence, they assume the probability that the client is randomly and uniformly selected or proportional to the size of the local data set. The problem of client characteristics varying over time throughout the day is introduced and the convergence of semi-cyclic SGD is studied in the research [32], in which multiple blocks of clients with different characteristics are sampled from regular cyclic patterns, such as day and night. Due to the heterogeneity of computing power, clients can perform different local steps. Periodic decentralized SGD (PD-SGD) is proposed and is proved to be able to allow multiple local updates happed based on decentralized SGD in Federated Learning [33]. Yu et al. recommend allowing each customer to maintain local momentum buffers and average local buffers and local model parameters per communication turn. This approach, while empirically improving the final accuracy of local SGD, doubles the cost per round of communication [34] A new technique, FedFast, is presented to accelerate distributed learning to achieve good accuracy for all users early in the training process by sampling from a different set of participating customers in each training round and applying an active aggregation approach to propagate the updated model to other customers. Authors demonstrate the effectiveness of their approach on various benchmark datasets and compare it with state of the art recommendation techniques [35].

### Federated learning for healthcare

Since FL is a general learning paradigm that eliminates data pooling requirements for AI model development, it has applications across multiple scenarios, especially the entire AI healthcare [36]. A FL-based privacy-aware and resource-saving collaborative learning protocol was introduced in [37] for an EHR analysis management system working with multiple hospital institutions and cloud servers, where each hospital runs neural network models with its own EHR with the help of cloud computing. In addition, an FL-based approach was proposed to predict hospitalizations in patients diagnosed with heart disease using their historical EHR. More specifically, health data from an EHR system consisting of patient smartphones and distributed hospitals is trained locally on demographic information such as age, gender and physical characteristics [6]. [38] proposed a FLT scheme for wearable health monitoring, in which smartphones and cloud servers cooperated to train and share CNN model for the identification of privacy-conscious human activities. A disease prediction method using FL with a large national health insurance data set of 99 medical sites (such as hospitals and clinical laboratories) distributed across 34 states in the United States is studied [39]. The data included EHR for diabetes, psychological disorders, and ischemic heart disease. The FL approach achieves competitive performance in terms of high accuracy and privacy by comparing with traditional methods such as centralized learning and local training without federation. also builds a FL-based health monitoring solution for analyzing patient outcomes from distributed hospital networks. Interestingly, each hospital created an entity called the Personalized Treatment Effect Estimator. Each estimator can be classified in each subgroup, where individual treatment outcomes include outcomes of patient characteristics, and site indicators are used to estimate overall treatment outcomes at coordination sites [40].

A combined brain imaging method is proposed to utilize MRI scans distributed across multiple clinical centers and institutions. In this case, through collaboration between the medical site and the central server, an FL model was derived to simulate an end-to-end framework for data standardization, confounding factor correction, and high-dimensional feature variability measurements [41]. To facilitate X-ray scanning in intelligent health care, a FL-based approach is proposed to support the diagnosis of acute neurological symptoms such as severe headache or loss of consciousness. Each hospital runs a CNN-based DenseNet1212 model that supports feature propagation, encourages feature reuse, and minimizes the number of neural parameters to train the X-ray image dataset provided by the North American Radiology Society [42]. In addition, a dynamic fusion-based FL method is proposed for CT scan image analysis, which can diagnose COVID-19 infection through customer participation and customer selection. First, each customer, such as a medical institution, makes a decision about whether to participate in the FL rotation based on the performance of the new training model. The central server also calculates the update time to determine which clients are allowed to update their local gradient [43]. FL also combines with deep learning to build a deep collaborative learning solution for detecting COVID-19 lung anomalies in CT. The internal data set was collected from a total of 75 confirmed COVID-19 patients at three local hospitals in Hong Kong for FL simulation and was then validated by data from mainland China and Germany [44].

Our work differs from the above methods because our dynamic node alignment aggregates weights of matching nodes for FL by ensuring the best matching nodes across different sites.

## Proposed approach

### Motivation

Instead of using fixed node matching, like FedAvg does, we propose to use dynamic node matching to find matching node between different sites, and then aggregate weights of matched nodes to calculate weight values of the global model. During the FL process, the sites will pass their local weight values to the center, so the center will carry out node matching before aggregating site weight values. Our idea is to use weight values of each node as a feature vector to find matching nodes. Because weight values of a neuron are associated to each features, for nodes at the same hidden layer, they will have same input space. This allows us to use weight values to find distance/similarity between nodes for matching.

**Fig 4:**
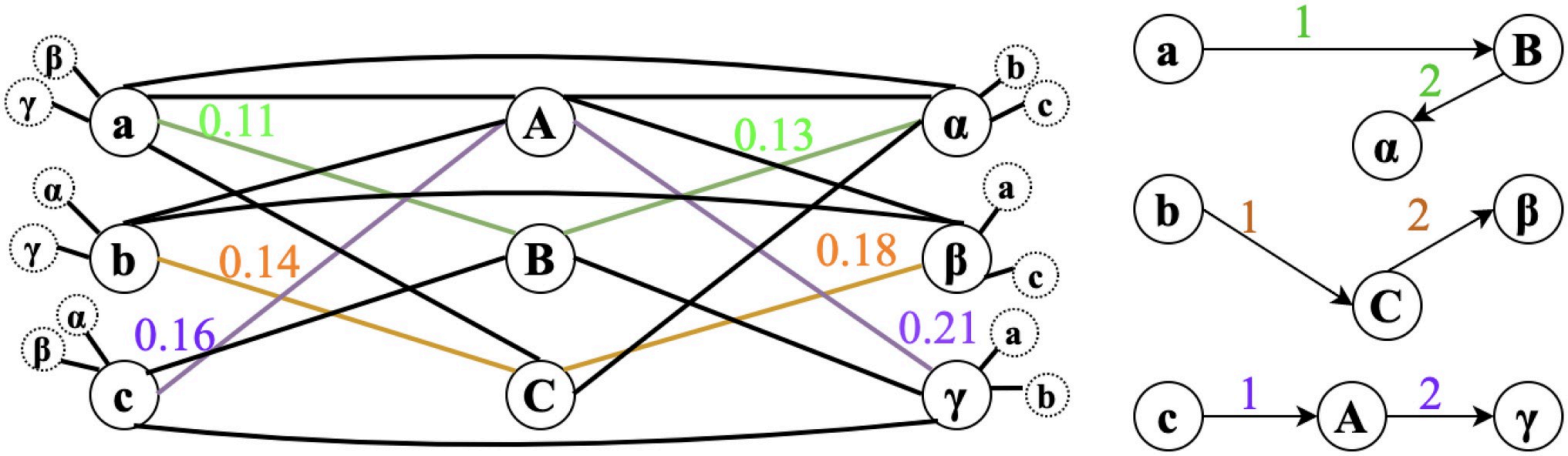
Node matching using MST. Node **a** is the starting point since it has the smallest distance 0.11 with node **B**, therefore, **B** will be matched to **a**. Node *α* will be matched with {**a**, **B**} with MST. This MST matching process will continue for node **b** and **c**.

To make sure weight values are aggregated from the most similar nodes crossing all clients *C*, at the first step, nodes distances are calculated across all clients as shown in the distance matrix in Table 2, from which Minimum Spanning Tree (MST) as shown in Fig 4 is used to ensure that the matching are across all clients. A minimum spanning tree (MST) or minimum weighting tree is a subset of edges of a connected edge-weighted undirected graph that joins all vertices together without any loops and with the smallest possible total edge weights. That is, it is a spanning tree with the smallest possible sum of edge weights. More generally, any edge-weighted undirected graph (not necessarily connected) has a minimum spanning forest, which is the union of the minimum spanning trees of its connected components [45]. In our example in Table 2, a distance mapping is plotted to demonstrate how the matching process works. At first node distances are calculated across all sites, in this case, 3 sites. we start the matching process from node *a* because it has the smallest distance 0.11 across all the nodes. we can observe that node *B* has the smallest distance with it, therefore, *B* will be matched to *a*. For the next step, we are using MST to find the next matching node for {*a*, *B*}, which in this case, will be node *α*. This MST matching process will continue until all the nodes are matched across all clients as shown in Fig 4.

**Table 2:** An example of pairwise distance tables between three sites where each site has three nodes: Site_1_={*A*, *B*, *C*}, Site_2_={*a*, *b*, *c*}, and Site_3_={*α*, *β*, *γ*}. Each value in the table denotes distance between two nodes across two sites.

	a	b	c
A	0.12	0.15	0.16
B	**0.11**	0.13	0.17
C	0.16	0.14	0.18
	A	B	C
*α*	0.27	0.13	0.19
*β*	0.23	0.14	0.18
*γ*	0.21	0.16	0.21
	a	b	c
*α*	0.13	0.24	0.18
*β*	0.14	0.19	0.21
*γ*	0.21	0.21	0.25

### Dynamic neural network node matching

In the proposed method, one key step is to find the closest nodes based on distance calculation in each round. This step is carried out at the center, and the aggregated weights are then dispatched to the federated learning site for the next round. The node matching is applied to one specific hidden layer of all networks, one at a time. By default, we are referring to nodes in the first hidden layer for ease of explanation. The same matching process is applicable to any other hidden layers as well. Algorithm 1 outlines the main steps of FedDNA for matching nodes across networks. Overall definition of the symbols used in our node matching is shown in Table 3.

**Table 3:** Definition for symbols used in node matching.

Symbol	Definition
**S**	Global model (server)
$v_{i}^{s}$	Weight vector of the *i*-th node in global model first hidden layer
C	Set of clients
**c** ^*α*^	Nodes weight vector of client **c**
$v_{i}^{\alpha }$	Node weight vector of client **c**^*α*^’s *i*-th node
$w_{i,d}^{\alpha }$	Weight values of node $v_{i}^{\alpha }$
*d*(*a*, *b*)	Distance between node *a* and node *b*
T	Minimum spanning tree
d(v,T)	Distance between node **v** and tree T

Denote $S=\{v_{1}^{s},v_{2}^{s},\ldots ,v_{n}^{s}\}$ the global model (server) in which $v_{i}^{s}=\left[w_{i,0}^{s},w_{i,1}^{s},\ldots ,w_{i,m}^{s}\right]$ is the weight vector of the *i*-th node in its first hidden layer. $C=\{c_{1},c_{2},\ldots ,c_{\Sigma }\}$ means the set of clients **c** and $c^{\alpha }=\left\{v_{1}^{\alpha },v_{2}^{\alpha },\ldots ,v_{n}^{\alpha }\right\}$ is nodes weight vector of client **c**^*α*^. Node weight vector of client **c**^*α*^’s *i*-th node is denoted by $v_{i}^{\alpha }=\left[w_{i,0}^{\alpha },w_{i,1}^{\alpha },\ldots ,w_{i,m}^{\alpha }\right]$.

#### Neuron matching distance calculation

Given two neurons $v_{i}^{\alpha }$ and $v_{j}^{\beta }$ at the same layer, because they have the same input dimensions (In this paper, we are using dense network architecture, so neurons at the same layer are connecting to all inputs/nodes of the preceding layer), we can represent each nuron as a vector, and calculate distance/similarity between neurons using the vectors.

Assume for any particular layer, the input dimension is *m*, and the weight values of neuron $v_{i}^{\alpha }=\left[w_{i,0}^{\alpha },w_{i,1}^{\alpha },\ldots ,w_{i,m}^{\alpha }\right]$, weight values of neuron $v_{j}^{\beta }=\left[w_{j,0}^{\beta },w_{j,1}^{\beta },\ldots ,w_{j,m}^{\beta }\right]$, respectively. Node distance between $v_{i}^{\alpha }$ and $v_{i}^{k}$ can be calculated with Euclidean distance defined in Eq 2 or using Manhattan distance defined Eq 3. The Euclidean distance between two points in Euclidean space is defined as the length of the line segment between the two points, which essentially represents the shortest distance between two points. Manhattan distance is a distance measure between two points in an *m*-dimensional vector space. It is the sum of the projected lengths of the line segments between the points on the coordinate axes. In simple terms, it is the sum of the absolute differences of two points measured in all dimensions.

$$\begin{array}{c} d_{Euclidean}\left(v_{i}^{\alpha },v_{j}^{\beta }\right)=\sqrt{\sum_{d=1}^{m}{\left(w_{i,d}^{\alpha }-w_{j,d}^{\beta }\right)}^{2}} \end{array}$$ (2)

$$\begin{array}{c} d_{Mahattan}\left(v_{i}^{\alpha },v_{j}^{\beta }\right)=\sum_{d=1}^{m}\left|w_{i,d}^{\alpha }-w_{j,d}^{\beta }\right| \end{array}$$ (3)

During the node matching process, we will be growing a tree (*i.e.* a minimum spanning tree MST) to link matched/aligned nodes across all sites. In this case, a tree T consists of a set of neurons, $i.e.,\;T=\{v_{i}^{\alpha },\ldots ,v_{j}^{\beta },\ldots \}$ where *α* ≠ *β*. We enforce *α* ≠ *β* such that an MST tree only contains one node from each site (because we are trying to find matching nodes across all sites. It does not make sense to have a neuron to match a node of the same network). The number of nodes in the tree T varies, as the tree is growing dynamically. However, after the matching, each node only belongs to one MST tree, and the final number of nodes in the MST tree equals to the number of sites of the FL learning framework. We do not record edges connecting nodes in the tree, because our goal is to find matching nodes as a group, and then use their weights to update center’s node weights. In this case, the pairwise relationship between sites is not important to us. Also, each tree T records its membership nodes and will use their weights to calculate the average weights, which will be pass to respective members of the tree T for next round FL learning.

During node matching, we need to expand the tree T and include matching node to the tree. Therefore, we define the distance between a node **v** and Minimum Spanning Tree T as in Eq 4. The distance from a node to a Minimum Spanning Tree tree argmind(v,T) equals to its distance to its closest node in the tree.

$$\begin{array}{c} d\left(v,T\right)=\underset{v^{\alpha }\;\in \;T}{\text{arg}}\text{min}d(v,v^{\alpha }) \end{array}$$ (4)

#### Minimum spanning tree for neuron alignment across sites

At the first step, each client downloads the model from central server and train it with its local data, after which client **c**^*α*^ is randomly chosen from C. One node $v_{i}^{\alpha }$ will be randomly selected among all the nodes in the first hidden layer of client **c**^*α*^’s local model. In the second step, another client **c**^*k*^ will be chosen at random from {C—**c**^*α*^}. A distance function explained previously will be used to calculate the distance $d(v_{j}^{k},v_{i}^{\alpha })$ between all nodes in the first hidden layer of client **c**^*k*^ model and node $v_{i}^{\alpha }$. We can get two nodes matched $\left(v_{j}^{k},v_{i}^{\alpha }\right)$ based on the smallest distance.

**Fig 5:**
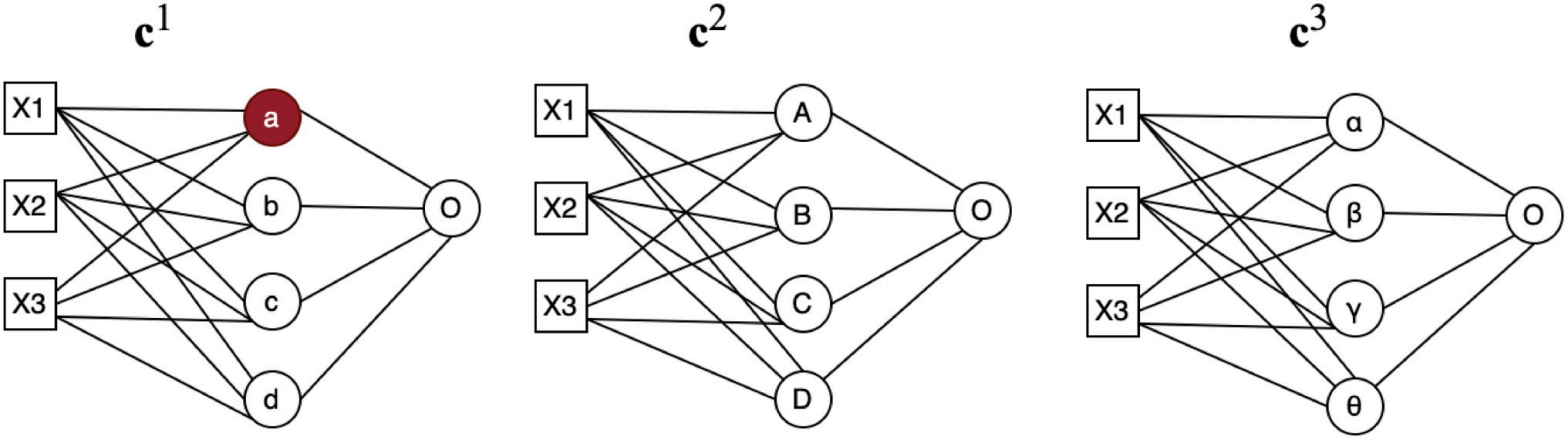
Node matching step 1. after each client finishing training its local model, client **c**^*α*^ is randomly chosen from C and node $v_{i}^{\alpha }$ will be randomly selected among all the nodes in the first hidden layer of client **c**^*α*^ local mode.

**Fig 6:**
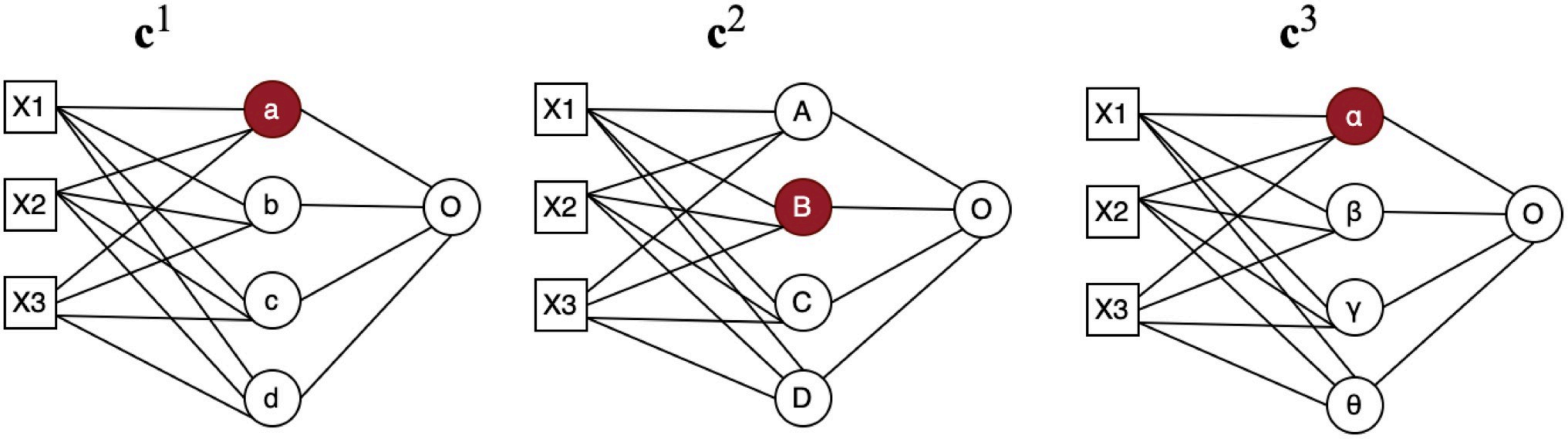
Node matching result. node {*a*, *B*, *α*} are matching nodes.

Now we have two nodes, which are also the start of our MST tree $T=\{v_{j}^{k},v_{i}^{\alpha }\}$, from which we will start to grow the tree. MST is the one whose cumulative edge weights have the smallest value, and in our proposed method, it means the one whose cumulative node distances have the smallest value. In each matching step, we will randomly pick one client from {C—{**c**^*α*^, **c**^*k*^}}. Node to tree distance Eq 4 will be applied to find the subsequent matching nodes to join the MST tree T. The MST tree T will continue growing until {C—{**c**^*α*^, **c**^*k*^,…}} is empty and at the same time, a complete tree T with new node set {$v_{i}^{\alpha }$, $v_{j}^{k}$, $v_{t}^{\beta }$,…} will be formed to aggregate their averaged weight values as a new node weight $v_{i}^{s}$ for the global model. To illustrated the above description, for example, one client is randomly chosen in Fig 5 then in Fig 6, after the first calculation, node {*a*, *B*} are matching node, then we calculate distance *d*(*a*, *α*), *d*(*a*, *β*), *d*(*a*, *γ*), *d*(*a*, *θ*), *d*(*B*, *α*), *d*(*B*, *β*), *d*(*B*, *γ*), *d*(*B*, *θ*), then choose node *α* with the smallest distance and node {*a*, *B*, *α*} are the matching nodes. Weight values {$v_{a}^{1},v_{B}^{2},v_{\alpha }^{3}$} will be averaged to be considered as a new node value for the global model.

**Algorithm 1:** FedDNA: Federated learning with dynamic node alignment

**Input:** *l*: The index of the layer to apply node alignment (default first hidden layer)

**Data:** Client models’ node weight values;

 Chosen node set N={};

 Matched node set M={};

 Chosen Client set C={};

**Output:** Aggregated weight values of the global model **S**

**1** C← Set of federated learning participating clients

**2** N← Node set of the *l*^*th*^ hidden layer of participating clients (C)

**3** **W** ← Obtain *l*^*th*^ layer’s weight values from participating clients (C)

**4** **c**^*α*^← Randomly select one client from the client set C

**5** {$v_{1}^{\alpha },v_{2}^{\alpha },\ldots ,v_{n}^{\alpha }\}\leftarrow$ Obtain client **c**^*α*^’s layer *l* node weight vectors

**6** M←∅;R←N ;    //Initialize matched node set (M) and remaining unmatched node set (R)

**7** $\bar{W}\leftarrow \emptyset$ ;    //Initialize set ($\bar{W}$) storing mean weight values of matched nodes across all sites

**8** **while** R *is not empty*;    //loop until all nodes are matched

**9** **do**

**10**  $C^{\prime }\leftarrow C$;    //A temporary set to ensure that each site has one node being matched, one at a time

**11**  **c**^*α*^← Randomly select one client from the client set C

**12**  $v_{i}^{\alpha }\leftarrow$ Randomly select on neuron of **c**^*α*^ from remaining node set R

**13**  $T\leftarrow \{v_{i}^{\alpha }\}$;    //Initialize MST tree for matching

**14**  $R\leftarrow R\backslash v_{i}^{\alpha }$;    //Exclude $v_{i}^{\alpha }$ from remaining node set R

**15**  $C^{\prime }\leftarrow C\backslash c^{\alpha }$;    //Exclude selected site **c**^*α*^, because its node already in the tree T

**16**  **while** $C^{\prime }$ *is not empty*;    //loop until all sites are matched

**17**  **do**

**18**  $\left[v^{*},\;c^{*}\right]\leftarrow \underset{v*\in c^{*};\;c^{*}\in C^{\prime }}{\text{arg}\;\text{min}}\;d\left(v^{*},T\right)$;    //find node v^*^ most closest to the MST tree T

**19**  $T\leftarrow T\cup v^{*}$;    //Include matched node v^*^ to the tree T

**20**  $C^{\prime }\leftarrow C^{\prime }\backslash c^{*}$;    //Exclude site c^*^

**21**  $R\leftarrow R\backslash v^{*}$;    //Exclude v^*^ from remaining node set R

**22**  **end**

**23**  $\bar{w}\leftarrow \text{Average}\left(v_{k}\right);\forall v_{k}\in T$;    //Calculate average weight values of matched notes in T

**24**  $\bar{W}\leftarrow \bar{W}\cup \bar{w}$;    //Center collects average weights of matched nodes across all sites

**25**  M←M∪T;    //Include all MST tree nodes to the matched set M

**26** **end**

**27** **for** each client $c^{\alpha }\;\in \;C$ **do**

**28**  **W** ← ClientUpdate$\left(c^{\alpha },\bar{W}\right)$;    //Dispatch mean weight values to each site for next round federated learning (Alg. 2)

**29**  **end**

**Algorithm 2:** ClientUpdate(c,**w**): Local client weight updating

**Input: w**: trainable model parameters;

**Data:** (1) *D*_*c*_: local data at site *c*; (2) *b*: batch size; (3) *e*: Number of epochs; (4) *η*: learning rate;

**Output: w**: updated local model parameters;

**1**  B← split local data *D*_*c*_ into batches of size *b*

**2**  **for** *each epoch from 1 to e* **do**

**3**  **for** *batch* b∈B **do**

**4**   **w** ← **w** − *η*▽*ι*(**w**;**b**)

**5**  **end**

**6** **end**

**7** Return **w**

#### Dynamic node alignment *vs*. Frozen

In our proposed method, frozen means instead of using dynamic node alignment through the entire training process, we choose to train the federated learning model with dynamic node alignment for certain rounds at the very first beginning, then static node alignment will be applied for the rest training part. By doing so, nodes with similar response will be paired right after the training process starts and once all the neurons are matched during the first certain rounds, we believe that the node pair pattern will discovered and fixed to a certain extend, therefore, using static node alignment will prevent the pattern from being disturbed from subsequent training process.

#### Theoretical analysis

In this subsection, we analyze the time complexity of FedDNA, and compare its complexity with simple global optimal matching search. Denote *Σ* the number of sites, *n* the number of first layer nodes at each site, and *m* the number of features for each neuron. Because all sites in FL setting have same network structure, we only focus on first layer, and the same analysis applies to other layers, if dynamic node alignment is carried out beyond the first layer.

Finding global optimal matching (*i.e.*, the sum of matching distances of all nodes, across all sites) is a combinatorial problem, because it requires comparisons of all nodes against all other nodes, across all sites. For two sites, each having *n* nodes, the matching complexity is O(n×n×m), because it needs to cross check all pairs (and each pairs involve *m* feature dimension comparisons). Adding a third site would result in O(n×n×n×m) complexity because all node pairs between three sites need to be checked. As a result, for *Σ* sites the total complexity is $O(n^{\Sigma }\times m)$, which grows exponentially with respect to the number of sizes.

For FedDNA, finding matching nodes across all sites for one node requires O((Σ-1)×n×m) complexity because a node needs to search all nodes from other sites and it does not need to search nodes from the same site. Once the first node is matched (across all sites) and matched nodes are added to the minimum spanning tree (MST), the next node matching requires O((Σ-1)×(n-1)×m) complexity because there are *n*−1 unmatched nodes remain for each site. As a result, total time complexity for all nodes (across all sites) is the sum of all individual nodes’ complexity: $O\left(\left(\Sigma -1\right)\times n\times m\right)+O\left(\left(\Sigma -1\right)\times \left(n-1\right)\times m\right)+\ldots +O\left(\left(\Sigma -1\right)\times 1\times m\right)=O\left(\Sigma \times n^{2}\times m\right)$. By growing minimum spanning tree (MST) to support the matching, FedDNA reduces the exponential complexity from $O(n^{\Sigma }\times m)$ (for global optimal matching) to quadratic $O(\Sigma \times n^{2}\times m)$.

In summary, FedDNA’s complexity is linear with respect to the number of sites, and quadratic with respect to the number of nodes at each site.

## Experiments

### Datasets

We used four benchmark datasets in the experiments. The first one is Diabetes Data Set which data source are obtained from two main aspects, an automatic electronic recording device and paper records to predict whether a patient has diabetes or not. For the automated electronic recording devices, they have an internal clock to mark events, whereas paper records only provide periods of “logical time” (breakfast, lunch, dinner, bedtime). For paper records, fixed times are assigned to breakfast (08:00), lunch (12:00), dinner (18:00) and bedtime (22:00). Therefore, paper records have a fictional uniform time of recording, while electronic records have a more real time stamp [46]. The second dataset we used is Spam_base Data Set from UCI which was created by spam emails from postmaster and individuals and non-spam emails from filed work and personal e-mails in order to construct a personalized spam filter. In this dataset, The last column indicates whether the email is considered spam (1) or not (0), that is, unsolicited commercial email. Most properties indicate whether a particular word or character occurs frequently in emails [47]. Another data set used in this paper is called Patient Survival Prediction Dataset. It uses knowledge about patient chronic conditions from Intensive Care Units (ICUs) to inform clinical decisions about patient care and ultimately predict patient’s survival outcomes [48]. Occupancy Detection Data Set is the last data set we used to verify our model’s performance. It is a dataset for predicting room occupancy using environmental factors such as Temperature, Humidity, Light and CO2. Ground-truth occupancy obtained from time stamped pictures that were taken every minute [49].

Basic descriptions about these four datasets are shown in Table 4 from which we can observe the number of samples in each dataset, diabetes database has 1150 samples and there are 4601, 91714 instances in the spambase data set and Patient Survival Prediction data set separately. Patient Survival Prediction set has the most samples and also the largest attributes while Occupancy detection data set has the medium size samples with the least number of attributes. Apart from that, data dimensions of those four are also different with various feature types such as categorical features, numerical features. One same point is that there are only two classes in all the datasets, which means binary classification will be performed in our proposed model.

**Table 4:** Summary of the benchmark datasets used in the experiments, including sample amount, attributes amount, data characteristics and class distribution.

Dataset	# of instances	# of attributes	Attribute Characteristics	Class	Class Distribution	Class set up
Diabetes Database	1,150	19	Categorical, Integer	Binary	0.89	0.2; 0.4; 0.5; 0.6; 0.7
Spambase Data Set	4,601	56	Integer, Real	Binary	1.54	0.8; 0.6; 0.5; 0.4; 0.2
Patient Survival Prediction	91,714	186	Categorical, Real	Binary	11.26	0.5; 1; 3; 5; 7
Occupancy Detection	20,560	7	Real	Binary	3.33	0.5; 1; 1.5; 2; 2.5

### Baseline methods

To validate the performance of the proposed method, we use deep neural networks as the training models and employ four baselines for our comparisons. One is plain neural network (Plain_NN) model which has the same structure as our proposed model which has one input layer, two hidden layers and one output layer.

#### FedAvg

The second baseline is called Federated Average (FedAvg), which also share the same network structure with our proposed method and use static node matching to aggregated node weight values for the global model. In FedAvg, each client downloads the current model from a central server, improves it by learning from its own local data, and then aggregates the changes into a small centralized update. Only updates to the model are sent to the server/cloud using encrypted communication and immediately averaged with other user updates to improve the shared model based on Eq 1. All training data is kept locally and no individual updates are stored in the cloud.

Federated Average (FedAvg) is a generalization of FedSGD that allows local nodes to perform multiple batch updates to local data and swap updated weights instead of gradients. The basic principle behind this generalization is that in FedSGD, if all local nodes start from the same initialization, the mean gradient is strictly equivalent to the mean weight itself. Furthermore, averaging adjustment weights from the same initialization does not necessarily harm the performance of the resulting averaging model [1, 7].

#### FedDyn

Next baseline is call FedDyn, in which each client learns a unique model with its own regularization parameter [50]. In this method, each client in the federated learning system learns a unique model with its own regularization parameter. The regularization parameter is updated dynamically during the training process based on the client’s local model performance. This means that clients with more difficult data can have a higher regularization, while clients with easier data can have a lower regularization, which improves the convergence speed and accuracy of the federated learning process.

The objective of FedDyn is to solve. Eq 5, where *k*Â ∈ [*m*] consists of *N*_*k*_ training instances, *L*_*k*_(*θ*) is the empirical loss of the *k*_*th*_ device and *θ* are the parameters of the neural network.

$$\begin{array}{c} arg\;\underset{\theta \in R^{d}}{\text{min}}\left[l\left(\theta \right)\right]≜\frac{1}{m}\sum_{k\in [m]}L_{k}\left(\theta \right) \end{array}$$ (5)

#### FedDNA_fixed_

Baseline 4 (FedDNA_fixed_) calculates nodes’ distance based on a fixed node. This baseline is created because we want to confirm whether the node matching pattern in dynamic node alignment improve compared with when the node used for matching remains the same. At the first step, after each client finishing training its local model, client **c**^*α*^ is randomly chosen from C. Then one node $v_{i}^{\alpha }$ will be randomly selected among all the nodes in the first hidden layer of client **c**^*α*^’s local model. In the next step, one client will be randomly **c**^*k*^ picked from {C—**c**^*α*^}, a distance function explained previously will be used to calculate the distance $d(v_{j}^{k},v_{i}^{\alpha })$ between all nodes in the first hidden layer of client **c**^*k*^ model and node $v_{i}^{\alpha }$. We can get two nodes matched $\left(v_{j}^{k},v_{i}^{\alpha }\right)$ based on the smallest distance.

Now we have two nodes, which are also the start of our MST tree $T=\{v_{j}^{k},v_{i}^{\alpha }\}$, from which we will start to grow the tree. In each matching step, we will randomly pick one client from {C—{**c**^*α*^, **c**^*k*^}}. Unlike FedDNA, in this baseline, the distance between a node to the tree will be calculated with Eq 6, which means that only $v_{i}^{\alpha }$ will be used in MST tree T to do the node matching. The MST tree T will continue growing until {C—{**c**^*α*^, **c**^*k*^,…}} is empty and at the same time, a complete tree T with new node set {$v_{i}^{\alpha }$, $v_{j}^{k}$, $v_{t}^{\beta }$,…} will be formed to aggregate their averaged weight values as a new node weight $v_{i}^{s}$ for the global model.

$$\begin{array}{c} d\left(v,T\right)=d_{\text{Euclidean}/\text{Manhattan}}\left(v,v_{i}^{\alpha }\right) \end{array}$$ (6)

For example, in Fig 5, distance for **c**^2^ will be *d*(*a*, *A*), *d*(*a*, *B*), *d*(*a*, *C*), *d*(*a*, *D*) and for **c**^3^ the distance will be *d*(*a*, *α*), *d*(*a*, *β*), *d*(*a*, *γ*), *d*(*a*, *θ*). Assume for **c**^2^, the smallest distance is *d*(*a*, *B*) and *d*(*a*, *α*)for **c**^3^, then node {$v_{a}^{1},v_{B}^{2},v_{\alpha }^{3}$} are the matching nodes and their weight values will be averaged as one new node weight values for the global model.

#### FedDNA_random_

The last baseline is a modification based on both FedDNA and FedDNA_fixed_. Instead of being too static or too dynamic with the node matching, we cant to confirm the feasibility when the matching node is neither 100% percent fixed nor using the entire MST tree as a matching node. Settings for baseline 4 (FedDNA_random_) is as follows: At the first step, after each client finishing training its local model, client **c**^*α*^ is randomly chosen from C. Then one node $v_{i}^{\alpha }$ will be randomly selected among all the nodes in the first hidden layer of client **c**^*α*^’s local model. In the next step, one client will be randomly **c**^*k*^ picked from {C—**c**^*α*^}, a distance function explained previously will be used to calculate the distance $d(v_{j}^{k},v_{i}^{\alpha })$ between all nodes in the first hidden layer of client **c**^*k*^ model and node $v_{i}^{\alpha }$. We can get two nodes matched $\left(v_{j}^{k},v_{i}^{\alpha }\right)$ based on the smallest distance.

Now we have two nodes, which are also the start of our MST tree $T=\{v_{j}^{k},v_{i}^{\alpha }\}$, from which we will start to grow the tree. In our third step, one node will be randomly chosen from {$v_{i}^{\alpha }$, $v_{j}^{k}$} which will be used to match nodes of client {C—{**c**^*α*^, **c**^*k*^}} using Eq 4. Step 3 will be repeated until {C—{**c**^*α*^, **c**^*k*^,…}}is empty and at the same time, a new node set {$v_{i}^{\alpha }$, $v_{j}^{k}$, $v_{t}^{\beta }$,…} will be formed to aggregate their averaged weight values as a new node weight value for the global model. Assume we randomly choose **c**^2^ in Fig 5 to do the first match, node {*a*, *B*} are the matching nodes, then one node will be randomly chosen from node {*a*, *B*} to calculate distance for **c**^3^. If node *B* is chosen, distance *d*(*B*, *α*), *d*(*B*, *β*), *d*(*B*, *γ*), *d*(*B*, *θ*), will be calculated to choose the next matching node.

### Experiments setting

Our overall experiment setting is shown in Table 5. We use 10-fold cross validation, under which there will be 10 training rounds for each model to train. For each dataset, our aim is to predict the corresponding target and 10-fold cross validation is applied to reduce both bias and variance. Under each cross validation fold *K*, same weight values are initialized for all both baseline models and our proposed models, *Plain*_*NN*, *FedAvg*, *FedDyn*, *Baseline*_3_, *Baseline*_4_ and *FedDNA*. For methods under FL setting, model parameters will be passed to each clients at the very beginning of training. Training data will be randomly split into 5 sites and distributed to 5 clients, which is able to training the local model using their own data, after which weight values will be aggregated based on different FL method and then send back to the global models. Global models will pass the new calculated parameters to their local clients to start new round training until the convergence. For our proposed method FedDNA, there are two experiment settings in this paper. One is called no-freezing weight update setting, in which weight values of the global model will be aggregated using FedDNA method for all the 10 rounds while the second type of setting is called freezing, in other word, we will choose to update the global model parameters with FedDNA at the first *i* round and after that FedAvg will be used to aggregate clients’ model weight values for the rest of rounds. We design this type of setting because we think the first several rounds of distance calculation will give use the answer of the closest matching nodes then we can use that match to directly aggregated the node weight values.

**Table 5:** The pseudo code of the experiment settings and comparisons (all methods are compared based based on same training/test data. The initial network weights of each site are the same for different methods to avoid discrepancy due to random weight initialization.

*Experiment setting: Node_matching Federated Learning*
**Input** (1) Datasets D; (2) Node matching setting: Freeze; No freeze
**Output** Prediction of the target *y*
**For** each cross validation fold *K*:
**Initialize** same weight values for all the global models:
*Plain*_*NN*, *FedAvg*, *FedDyn*, (*FedDNA*_*fixed*_), (*FedDNA*_*random*_) and *FedDNA*
**Split** training data into 5 sites for 5 clients
**For** *Baseline*_1_:
Train model using all the training data
**For** federated learning models:
Client train their own model using their own data
Match nodes with their distance calculation principle
Aggregated weight values pass to each global model
**End For**
**Evaluate** each global models

For our experiment dataset settings, we first run our model based on the original class distributions across all clients in all datasets which is *negative*: *positive* = 1.54 in spam database, *negative*: *positive* = 0.89 in diabetes data base, *negative*: *positive* = 11.26 in Patient Survival Prediction data set and *negative*: *positive* = 3.33 in Occupancy Detection data set. In the second experiment setting, for each training process, 2 clients are randomly chosen to exchange 2/3 of their data while the rest 3 clients keep their own data, in this case, our model will be verified on non-IID datasets. Calculated overall node distance, Accuracy, AUC, F1_score, Balanced accuracy and Loss are used as performance measurement metrics.

Apart from randomly selecting 2 clients to exchange their data, we decide to evaluate our proposed model under different class distribution settings. The original class distribution (negative:positive) of the four datasets are as shown in Table 4. A set of class distributions is set up for the original four data sets to check the model performance. Since all the datasets have different original class distributions, the assigned class distributions of the four datasets in this paper are different from each other.

### Overall model performance

Tables 6–9 show the results for Diabetes dataset, Spam dataset, Occupancy data set and Patient survival data set respectively in our first dataset setting. Due to page limitation, only the best model performance results are presented in this paper. For Diabetes database, we can observe that FedDNA, which uses our proposed method FedDNA is able to find nodes combinations where the total node distance is the smallest with value 42.1352 compared with other methods whose final distance results are greater than 50. At the same time, FedDNA presents better metrics performance. Similarly, the smallest overall node distance and better metric performance are realized by our FedDNA method for spam database. However, we can also come to the conclusion that smaller overall node distance and better metrics performance does not always come with the smallest training loss, especially for FedDNA. For occupancy and patient survival prediction datasets, FedDNA shows similar performance as for the previous two datasets. Its overall classification performance outperforms all the baselines with the smallest node distance 5.7316 and 57.4096 respectively after matching, which indicates FedDNA is able to pair closest nodes together. We can tell that overall, for all the four datasets, when class distributions are the same across all clients, our proposed method performs the best in the freezing setting when the first two rounds using Manhattan distance to find the matching nodes and the rest using FedAvg with the smallest overall distance under freeze first two rounds experiment setting with 42.1352 for diabetes dataset, 730.3930 for the spam data set, 5.7316 for Occupancy Detection and 57.4096 for Patient Survival Prediction data set.

**Table 6:** Experimental results from Diabetes dataset using Manhattan distance based matching. For FedDNA, the matching freezes after first two rounds of dynamic node alignment.

	Distance	Accuracy	AUC	F1_score	Balanced accuracy	Loss
*Plain*_*NN*		0.6755	0.7529	0.6361	0.6744	3.3221
*FedAvg*	51.7421	0.7165	0.8044	0.7029	0.7219	1.9016
*FedDyn*	53.0637	0.7016	0.8012	0.7147	0.7078	1.8932
*FedDNA*_*fixed*_	63.7216	0.7264	0.7961	0.7120	0.7341	1.8354
*FedDNA*_*random*_	56.3497	0.7298	0.8003	0.7280	0.7396	1.8274
*FedDNA*	**42.1352**	**0.7381**	**0.8230**	**0.7290**	**0.7434**	**1.9253**

**Table 7:** Experimental results from Spam dataset using Manhattan distance based matching. For FedDNA, the matching freezes after first two rounds of dynamic node alignment.

	Distance	Accuracy	AUC	F1_score	Balanced accuracy	Loss
*Plain*_*NN*		0.9165	0.9536	0.8905	0.9095	0.5110
*FedAvg*	824.3761	0.9320	0.9717	0.9120	0.9294	0.4823
*FedDyn*	792.1406	0.9316	0.9719	0.9136	0.9117	0.4431
*FedDNA*_*fixed*_	798.0362	0.9350	0.9762	0.9187	0.9343	0.6352
*FedDNA*_*random*_	911.6532	0.9351	0.9772	0.9178	0.9337	0.4521
*FedDNA*	**730.3930**	**0.9376**	**0.9781**	**0.9210**	**0.9357**	**0.4841**

**Table 8:** Experimental results from Occupancy detection dataset using Manhattan distance based matching. For FedDNA, the matching freezes after first two rounds of dynamic node alignment.

	Distance	Accuracy	AUC	F1_score	Balanced accuracy	Loss
*Plain*_*NN*		0.8327	0.9578	0.5018	0.7021	1.4599
*FedAvg*	7.8736	0.9225	0.9715	0.8236	0.8910	1.1592
*FedDyn*	8.5130	0.9135	0.9713	0.8137	0.8862	1.0927
*FedDNA*_*fixed*_	10.4510	0.9346	0.9751	0.8681	0.9179	0.9031
*FedDNA*_*random*_	9.6437	0.9306	0.9762	0.8699	0.9083	0.8621
*FedDNA*	**5.7316**	**0.9402**	**0.9788**	**0.8710**	**0.9140**	**0.8711**

**Table 9:** Experimental results from Patient survival prediction dataset using Manhattan distance based matching. For FedDNA, the matching freezes after first two rounds of dynamic node alignment.

	Distance	Accuracy	AUC	F1_score	Balanced accuracy	Loss
*Plain*_*NN*		0.8201	0.5309	0.0618	0.4998	1.7521
*FedAvg*	63.2566	0.7913	0.6185	0.0676	0.4942	1.1150
*FedDyn*	67.9825	0.7740	0.6098	0.0635	0.5099	1.0047
*FedDNA*_*fixed*_	71.2609	0.9083	0.6422	0.0302	0.5024	1.0670
*FedDNA*_*random*_	75.6094	0.8633	0.6251	0.0232	0.5075	1.0427
*FedDNA*	**57.4096**	**0.8898**	**0.6485**	**0.0297**	**0.5079**	**1.4629**

### FedDNA *vs*. FedAvg with respect to different class distributions

**Fig 7:**
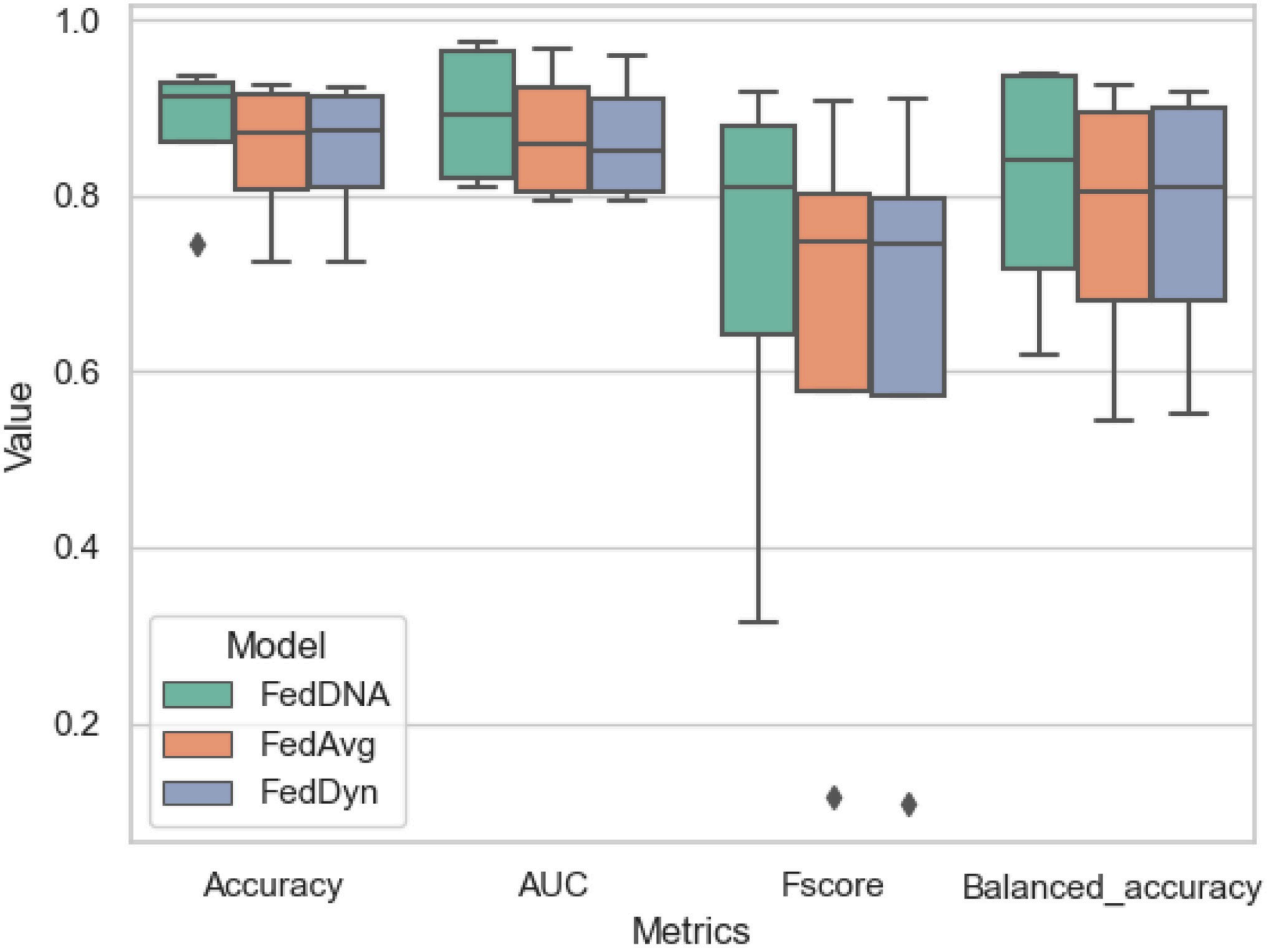
Overall performance comparisons between FedDNA and FedAvg, FedDyn. Outliers ca be observed from both methods, overall FedDNA outperforms FedAvg and FedAvg performs similarly as FedDyn.

Fig 7 is a box-plot for our second experiment setting’s results. Instead of showing all the results of all models across all the datasets, since results from the first setting highlights that FedDNA outperforms FedAvg and FedDyn overall across all the datasets, only comparisons between FedDNA and FedAvg, FedDyn with the combined results across all the datasets are shown in Fig 7, in which outliers can be observed for three models but overall we can come to the conclusion that when data is not evenly distributed across all clients, FedDNA performs the best in the freezing setting when the first two rounds using Manhattan distance to find the matching nodes and the rest using FedAvg.

**Fig 8:**
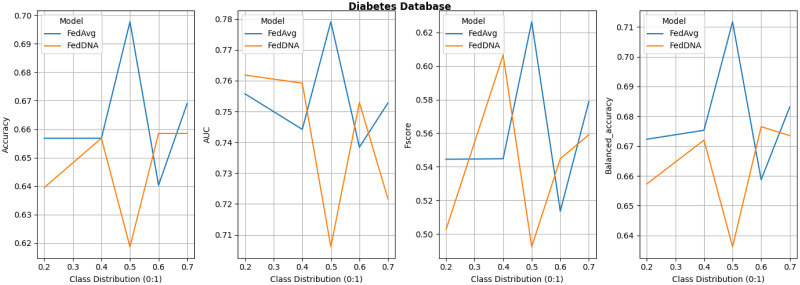
Performance comparisons between FedDNA and FedAvg with respect to different class distributions for diabetes dataset.

**Fig 9:**
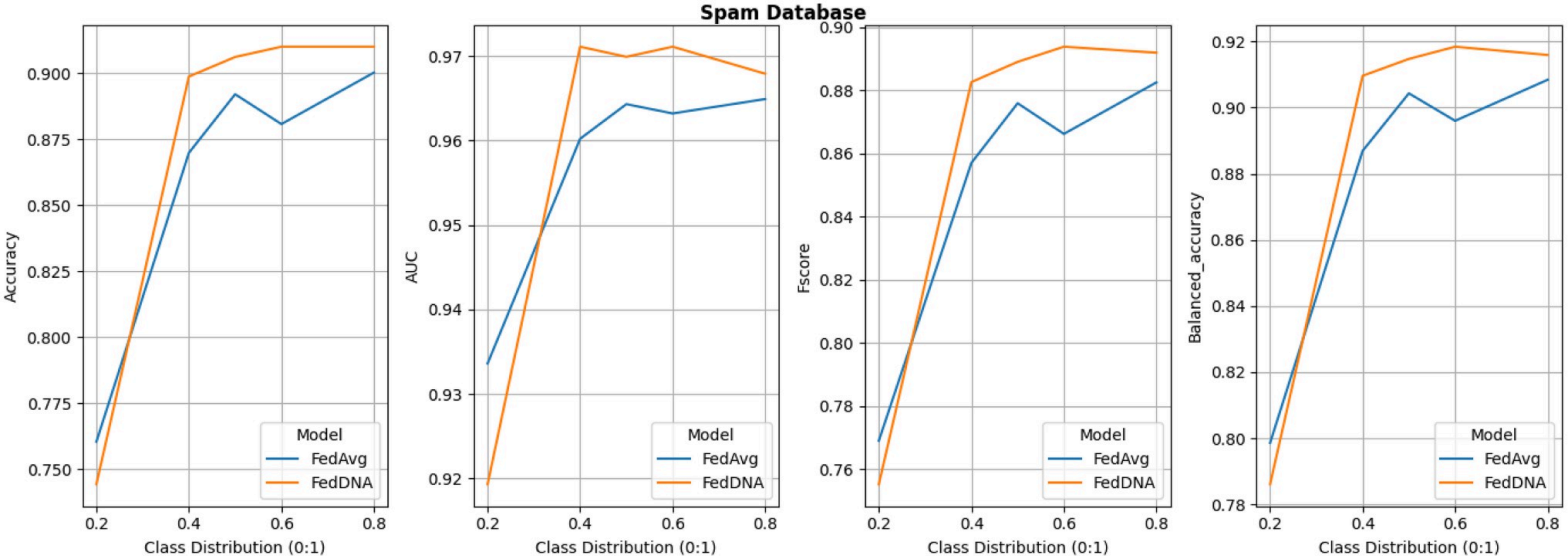
Performance comparisons between FedDNA and FedAvg with respect to different class distributions for spam dataset.

**Fig 10:**
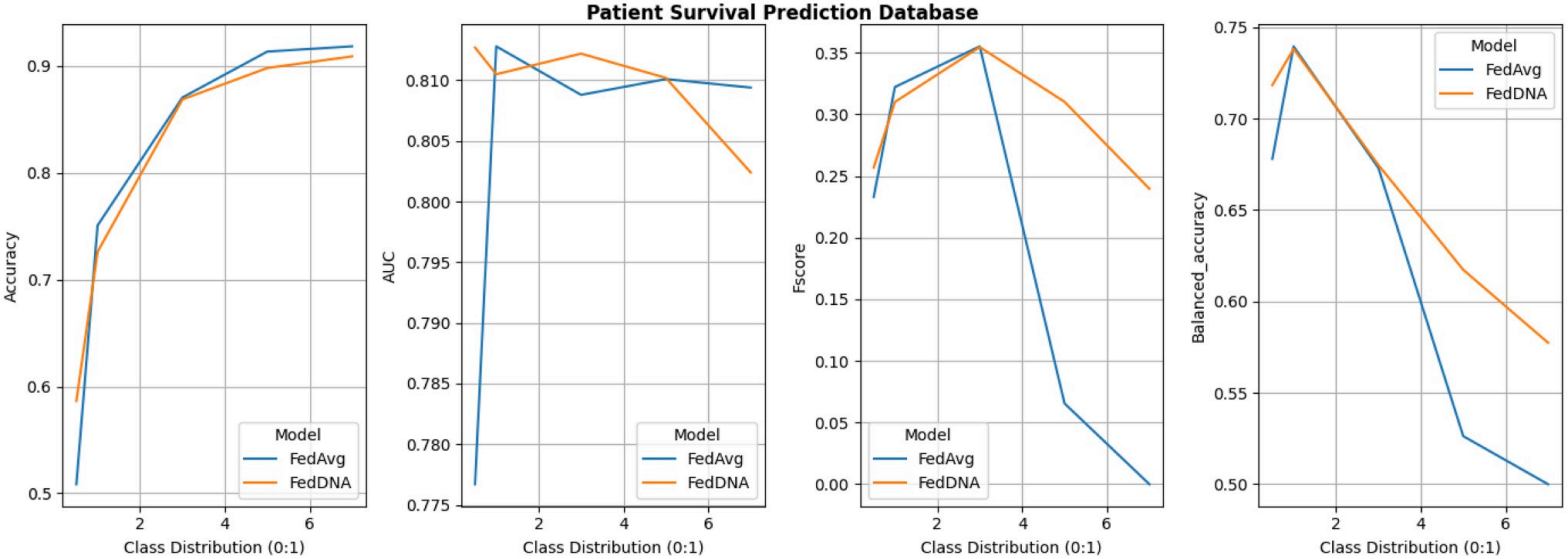
Performance comparisons between FedDNA and FedAvg with respect to different class distributions for patient survival prediction dataset.

**Fig 11:**
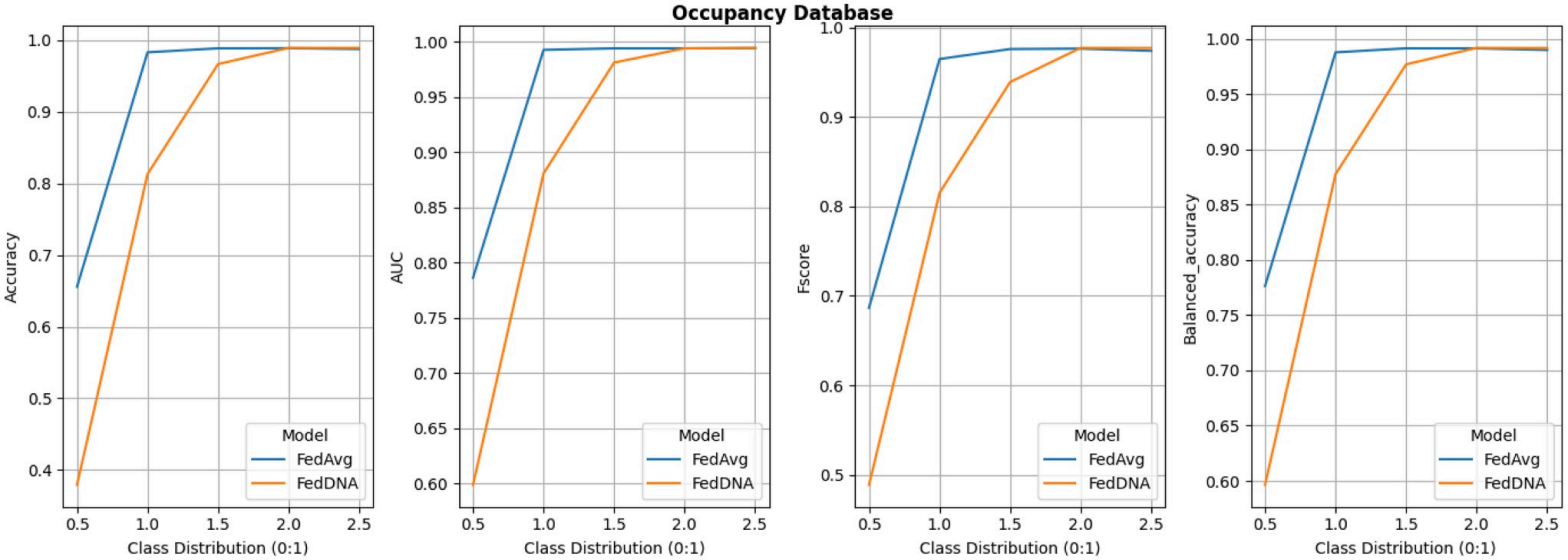
Performance comparisons between FedDNA and FedAvg with respect to different class distributions for occupancy dataset.

Since under this experiment setting, FedDyn does not deliver better overall performance than FedAvg according to above tables and figure, its detailed comparison with FedDNA is not demonstrated. Figs 8–11 report the performance of FedDNA and FedAvg, with respt to different class distributions (the class distributions were adjusted to assess the algorithm performance under different conditions). The *y*-axis is the values of each measurement and *x*-axis is different class distribution set ups for each dataset as shown in Table 4.

For Diabetes Dataset, FedDNA and FedAvg has the largest gap for all the measurements when sampling rate is 0.5 and both models’ performance fluctuate a lot with the change of class distributions. For Spam Dataset, even though FedDNA and FedAvg perform similarly when class distribution is smaller, as more and more negative samples shown in the datasets, FedDNA starts to show more advantages than FedAvg, especially when negative instances take up more than 40% of the dataset, the gap between both models become larger with a better performance from FedDNA. Similarly, for Patient Survival Prediction Dataset, the larger the sampling rate is, the better FedDNA outperforms FedAvg especially in terms of Fscore and Balanced accuracy. While for Occupancy dataset, FedDNA does not show much better results than FedAvg when class distribution is less than 2, after which both models the performance of the two models tends to be consistent.

## Discussion

In this paper, we propose a dynamic node matching method for federated learning. We argued that neural networks are inherently non-transparent and unstable, and the same network structure may end up with very different weight values, even with the same training data and same parameter settings. Traditionally, existing methods, such as FedAvg, force neurons across sites to be matched with predefined order, and use fixed matching nodes during the FL learning process. Alternatively, we proposed a dynamic node alignment, FedDNA, approach which dynamically finds matching nodes across sites, and uses matched nodes to calculate weight for FL learning. FedDNA represents each neuron as a vector, using their weight values, and calculate distances between neurons to find matching nodes. Meanwhile, because finding marching nodes are computationally expensive, we proposed a minimum spanning tree (MST) based approach to speed up the matching, with matched nodes across all sites being linked by using an MST tree. So the matching process is simply the MST tree growing process. Experiments and comparisons, including biased sample distributions, validate the performance of the FedDNA, compared to other baseline.

Future study can emphasize on the following three directions. First, we only studied dense networks and verified its performance using FedDNA. Future study can try to explore node matching between different types of network architectures, such as convectional nueral networks. Second, we only studied the proposed design using binary classification problems. In the future, multi-class classification problem will be explored using our proposed FedDNA method. For the last direction, we will use non-IID datasets to further adjust our model so that it can be applied not only to binary classification problem but also can achieve good results for datasets with different settings.

## References

[1] McMahan B, Moore E, Ramage D, Hampson S, y Arcas BA. Communication-efficient learning of deep networks from decentralized data. In: Artificial intelligence and statistics. PMLR; 2017. p. 1273–1282.

[2] Antunes RS, André da Costa C, Küderle A, Yari IA, Eskofier B. Federated Learning for Healthcare: Systematic Review and Architecture Proposal. ACM TIST. 2022;13(4):1–23. doi: 10.1145/3501813

[3] Xu J, Glicksberg BS, Su C, Walker P, Bian J, Wang F. Federated learning for healthcare informatics. J Healthc Inform Res. 2021;5(1):1–19. doi: 10.1007/s41666-020-00082-4 33204939 PMC7659898

[4] Qayyum A, Ahmad K, Ahsan MA, Al-Fuqaha A, Qadir J. Collaborative federated learning for healthcare: Multi-modal covid-19 diagnosis at the edge. IEEE Open Journal of the Computer Society. 2022;3:172–184. doi: 10.1109/OJCS.2022.3206407

[5] Nguyen DC, Pham QV, Pathirana PN, Ding M, Seneviratne A, Lin Z, et al. Federated learning for smart healthcare: A survey. ACM Computing Surveys (CSUR). 2022;55(3):1–37. doi: 10.1145/3501296

[6] Brisimi TS, Chen R, Mela T, Olshevsky A, Paschalidis IC, Shi W. Federated learning of predictive models from federated electronic health records. International journal of medical informatics. 2018;112:59–67. doi: 10.1016/j.ijmedinf.2018.01.007 29500022 PMC5836813

[7] Rieke N, Hancox J, Li W, Milletari F, Roth HR, Albarqouni S, et al. The future of digital health with federated learning. NPJ digital medicine. 2020;3(1):1–7. doi: 10.1038/s41746-020-00323-1 33015372 PMC7490367

[8] CDC. Health Insurance Portability and Accountability Act of 1996 (HIPAA); 1996. cdc.gov/phlp/publications/topic/hipaa.html.

[9] Verbraeken J, Wolting M, Katzy J, Kloppenburg J, Verbelen T, Rellermeyer JS. A survey on distributed machine learning. Acm computing surveys (csur). 2020;53(2):1–33. doi: 10.1145/3377454

[10] Li S, Cheng Y, Liu Y, Wang W, Chen T. Abnormal client behavior detection in federated learning. arXiv preprint arXiv:191009933. 2019;.

[11] Enthoven D, Al-Ars Z. Fidel: Reconstructing private training samples from weight updates in federated learning. arXiv preprint arXiv:210100159. 2021;.

[12] Deng Y, Kamani MM, Mahdavi M. Distributionally robust federated averaging. Adv Neural Inf Process Syst. 2020;33:15111–15122.

[13] Sun T, Li D, Wang B. Decentralized federated averaging. IEEE Transactions on Pattern Analysis and Machine Intelligence. 2022;. 10.1109/TPAMI.2022.3196503 35925850

[14] Yu F, Zhang W, Qin Z, Xu Z, Wang D, Liu C, et al. Fed2: Feature-aligned federated learning. In: Proceedings of the 27th ACM SIGKDD conference on knowledge discovery & data mining; 2021. p. 2066–2074.

[15] Wang H, Yurochkin M, Sun Y, Papailiopoulos D, Khazaeni Y. Federated learning with matched averaging. arXiv preprint arXiv:200206440. 2020;.

[16] Long G, Xie M, Shen T, Zhou T, Wang X, Jiang J. Multi-center federated learning: clients clustering for better personalization. World Wide Web. 2023;26(1):481–500. doi: 10.1007/s11280-022-01046-x

[17] Huang L, Yin Y, Fu Z, Zhang S, Deng H, Liu D. LoAdaBoost: Loss-based AdaBoost federated machine learning with reduced computational complexity on IID and non-IID intensive care data. Plos one. 2020;15(4):e0230706. doi: 10.1371/journal.pone.0230706 32302316 PMC7164603

[18] Liu D, Miller T, Sayeed R, Mandl KD. Fadl: Federated-autonomous deep learning for distributed electronic health record. arXiv preprint arXiv:181111400. 2018;.

[19] Haddadpour F, Kamani MM, Mokhtari A, Mahdavi M. Federated learning with compression: Unified analysis and sharp guarantees. In: AISTATS; 2021. p. 2350–2358.

[20] Bhagoji AN, Chakraborty S, Mittal P, Calo S. Analyzing federated learning through an adversarial lens. In: ICML; 2019. p. 634–643.

[21] Uddin MP, Xiang Y, Lu X, Yearwood J, Gao L. Mutual information driven federated learning. IEEE Trans Parallel Distrib Syst. 2020;32(7):1526–1538.

[22] Luo S, Chen X, Wu Q, Zhou Z, Yu S. HFEL: Joint edge association and resource allocation for cost-efficient hierarchical federated edge learning. IEEE Transactions on Wireless Communications. 2020;19(10):6535–6548. doi: 10.1109/TWC.2020.3003744

[23] Kang J, Xiong Z, Niyato D, Zou Y, Zhang Y, Guizani M. Reliable federated learning for mobile networks. IEEE Wireless Communications. 2020;27(2):72–80. doi: 10.1109/MWC.001.1900119

[24] Xu C, Liu S, Yang Z, Huang Y, Wong KK. Learning rate optimization for federated learning exploiting over-the-air computation. IEEE Journal on Selected Areas in Communications. 2021;39(12):3742–3756. doi: 10.1109/JSAC.2021.3118402

[25] Zheng C, Liu S, Huang Y, Zhang W, Yang L. Unsupervised Recurrent Federated Learning for Edge Popularity Prediction in Privacy-Preserving Mobile-Edge Computing Networks. IEEE Internet of Things Journal. 2022;9(23):24328–24345. doi: 10.1109/JIOT.2022.3189055

[26] Stich SU. Local SGD converges fast and communicates little. arXiv preprint arXiv:180509767. 2018;.

[27] Malinovskiy G, Kovalev D, Gasanov E, Condat L, Richtarik P. From local SGD to local fixed-point methods for federated learning. In: ICML; 2020. p. 6692–6701.

[28] Woodworth BE, Wang J, Smith A, McMahan B, Srebro N. Graph oracle models, lower bounds, and gaps for parallel stochastic optimization. Adv Neural Inf Process Syst. 2018;31.

[29] Wang J, Liu Q, Liang H, Joshi G, Poor HV. Tackling the objective inconsistency problem in heterogeneous federated optimization. Adv Neural Inf Process Syst. 2020;33:7611–7623.

[30] Khaled A, Mishchenko K, Richtárik P. First analysis of local gd on heterogeneous data. arXiv preprint arXiv:190904715. 2019;.

[31] Li X, Huang K, Yang W, Wang S, Zhang Z. On the convergence of fedavg on non-iid data. arXiv preprint arXiv:190702189. 2019;.

[32] Eichner H, Koren T, McMahan B, Srebro N, Talwar K. Semi-cyclic stochastic gradient descent. In: ICML; 2019. p. 1764–1773.

[33] Wang J, Joshi G. Cooperative sgd: A unified framework for the design and analysis of local-update sgd algorithms. The Journal of Machine Learning Research. 2021;22(1):9709–9758.

[34] Yu H, Jin R, Yang S. On the linear speedup analysis of communication efficient momentum SGD for distributed non-convex optimization. In: ICML; 2019. p. 7184–7193.

[35] Muhammad K, Wang Q, O’Reilly-Morgan D, Tragos E, Smyth B, Hurley N, et al. Fedfast: Going beyond average for faster training of federated recommender systems. In: ACM SIGKDD; 2020. p. 1234–1242.

[36] Li Y, Wang X, Zeng R, Donta PK, Murturi I, Huang M, et al. Federated Domain Generalization: A Survey. arXiv preprint arXiv:230601334. 2023;.

[37] Hao M, Li H, Xu G, Liu Z, Chen Z. Privacy-aware and resource-saving collaborative learning for healthcare in cloud computing. In: ICC 2020-2020 IEEE International Conference on Communications (ICC); 2020. p. 1–6.

[38] Chen Y, Qin X, Wang J, Yu C, Gao W. Fedhealth: A federated transfer learning framework for wearable healthcare. IEEE Intelligent Systems. 2020;35(4):83–93. doi: 10.1109/MIS.2020.2988604

[39] Liu D, Fox K, Weber G, Miller T. Confederated machine learning on horizontally and vertically separated medical data for large-scale health system intelligence. arXiv preprint arXiv:191002109. 2019;.

[40] Tan X, Chang CCH, Zhou L, Tang L. A tree-based model averaging approach for personalized treatment effect estimation from heterogeneous data sources. In: International Conference on Machine Learning. PMLR; 2022. p. 21013–21036. PMC10711748 38084268

[41] Silva S, Gutman BA, Romero E, Thompson PM, Altmann A, Lorenzi M. Federated learning in distributed medical databases: Meta-analysis of large-scale subcortical brain data. In: 2019 IEEE 16th international symposium on biomedical imaging (ISBI 2019). IEEE; 2019. p. 270–274.

[42] Srivastava UC, Upadhyay D, Sharma V. Intracranial hemorrhage detection using neural network based methods with federated learning. arXiv preprint arXiv:200508644. 2020;.

[43] Zhang W, Zhou T, Lu Q, Wang X, Zhu C, Sun H, et al. Dynamic-fusion-based federated learning for COVID-19 detection. IEEE Internet of Things Journal. 2021;8(21):15884–15891. doi: 10.1109/JIOT.2021.3056185 35663640 PMC9128757

[44] Dou Q, So TY, Jiang M, Liu Q, Vardhanabhuti V, Kaissis G, et al. Federated deep learning for detecting COVID-19 lung abnormalities in CT: a privacy-preserving multinational validation study. NPJ digital medicine. 2021;4(1):1–11. doi: 10.1038/s41746-021-00431-6 33782526 PMC8007806

[45] Graham RL, Hell P. On the History of the Minimum Spanning Tree Problem. Annals of the History of Computing. 1985;7(1):43–57. doi: 10.1109/MAHC.1985.10011

[46] Kahn M. Diabetes Data Set; 1994. https://archive.ics.uci.edu/ml/datasets/diabetes.

[47] Hopkins M, Reeber E, Forman G, Suermondt J. Spambase Data Set; 1999. https://archive.ics.uci.edu/ml/datasets/spambase.

[48] Raffa JD, Johnson AE, O’Brien Z, Pollard TJ, Mark RG, Celi LA, et al. The Global Open Source Severity of Illness Score (GOSSIS). Crit Care Med. 2022;50(7):1040–1050. doi: 10.1097/CCM.0000000000005518 35354159 PMC9233021

[49] Candanedo LM, Feldheim V. Accurate occupancy detection of an office room from light, temperature, humidity and CO2 measurements using statistical learning models. Energy and Buildings. 2016;112:28–39. doi: 10.1016/j.enbuild.2015.11.071

[50] Acar DAE, Zhao Y, Navarro RM, Mattina M, Whatmough PN, Saligrama V. Federated learning based on dynamic regularization. arXiv preprint arXiv:211104263. 2021;.

